# Utilizing human cerebral organoids to model breast cancer brain metastasis in culture

**DOI:** 10.1186/s13058-024-01865-y

**Published:** 2024-07-01

**Authors:** Chenran Wang, Aarti Nagayach, Harsh Patel, Lan Dao, Hui Zhu, Amanda R. Wasylishen, Yanbo Fan, Ady Kendler, Ziyuan Guo

**Affiliations:** 1 Department of Cancer Biology, University of Cincinnati College of Medicine, Cincinnati, OH 45267 USA; 2 Department of Pathology and Laboratory Medicine, University of Cincinnati College of Medicine, Cincinnati, OH 45267 USA; 3 Division of Developmental Biology, Cincinnati Children’s Hospital Medical Center, Cincinnati, OH 45229 USA

**Keywords:** Breast cancer, Brain metastasis, Cell-cell communication, Cerebral organoids, Neural cells, Tumor microenvironment

## Abstract

**Background:**

Metastasis, the spread, and growth of malignant cells at secondary sites within a patient’s body, accounts for over 90% of cancer-related mortality. Breast cancer is the most common tumor type diagnosed and the leading cause of cancer lethality in women in the United States. It is estimated that 10–16% breast cancer patients will have brain metastasis. Current therapies to treat patients with breast cancer brain metastasis (BCBM) remain palliative. This is largely due to our limited understanding of the fundamental molecular and cellular mechanisms through which BCBM progresses, which represents a critical barrier for the development of efficient therapies for affected breast cancer patients.

**Methods:**

Previous research in BCBM relied on co-culture assays of tumor cells with rodent neural cells or rodent brain slice ex vivo. Given the need to overcome the obstacle for human-relevant host to study cell-cell communication in BCBM, we generated human embryonic stem cell-derived cerebral organoids to co-culture with human breast cancer cell lines. We used MDA-MB-231 and its brain metastatic derivate MDA-MB-231 Br-EGFP, other cell lines of MCF-7, HCC-1806, and SUM159PT. We leveraged this novel 3D co-culture platform to investigate the crosstalk of human breast cancer cells with neural cells in cerebral organoid.

**Results:**

We found that MDA-MB-231 and SUM159PT breast cancer cells formed tumor colonies in human cerebral organoids. Moreover, MDA-MB-231 Br-EGFP cells showed increased capacity to invade and expand in human cerebral organoids.

**Conclusions:**

Our co-culture model has demonstrated a remarkable capacity to discern the brain metastatic ability of human breast cancer cells in cerebral organoids. The generation of BCBM-like structures in organoid will facilitate the study of human tumor microenvironment in culture.

## Introduction

Metastasis, the uncontrolled growth of malignant cells at secondary sites within a patient’s body, accounts for more than 90% of cancer-related mortality [1]. Breast cancer is the most common cancer in women and the leading cause for lethality in cancer patients in the United States [2]. It is estimated that 10–16% of breast cancer patients will have brain metastasis [3]. In addition, autopsy studies have demonstrated another 10% which were asymptomatic and therefore not diagnosed [4]. The incidence of breast cancer brain metastasis (BCBM) at recurrences is rising, which is likely due to prolonged survival of patients receiving more efficient treatments for primary disease and the availability of better imaging techniques that lead to increased detection of early brain metastases [5]. The risk of BCBM is correlated with breast cancer subtypes. Patients with triple negative breast cancer (TNBC) or Her2^+^ breast cancer subtypes experience higher brain metastasis occurrence than patients with luminal-like disease [3, 5, 6]. A study of metastatic TNBC indicates an estimated risk as high as 46% of brain metastasis in patients [7]. Unfortunately, all current therapies are merely palliative for BCBM [8]. One of the reasons is that the basic research on BCBM is far lagging to understand the molecular signaling pathways and the potential mechanisms for their resistance to the current therapies, owing in part to limited human-relevant experimental models of BCBM.

The development of BCBM is a complex process [9, 10]. A group of single or clustered breast cancer cells invade blood stream to circulate in the whole body. Once these circulating breast cancer cells reach the brain, a portion of them extravasate and enter brain. One of the most critical factors is the colonization and growth of breast cancer cells in the new tumor microenvironment (TME) of brain parenchyma [11, 12]. Targeting the TME is a potential therapeutic strategy to control BCBM. The breast cancer cells hijack resident and infiltrating non-tumor cells of the TME to promote their own growth, resist therapy, and suppress the immune system to prevent detection and elimination [13–15]. Disabling these interactions or reversing the impact on TME may outperform therapies that target the tumor itself which can gain resistance via mechanisms including heterogeneity and genomic instability [16]. Studies of bidirectional communication between breast cancer cells and TME cells will yield previously unknown tumor supporting interactions and new, effective therapeutic or preventive targets for BCBM.

To better understand the communication between cancer cells and new TME, 2D co-culture models for cancer cells growing with brain cells, and organotypic brain slices ex vivo have been used [17], which advance our understanding of their cell-cell communication. However, a major limitation for most, if not all these models is that the hosts originate from animals with significant differences in genetic background. Brain organoids are self-organizing CNS-like structures that form from embryonic stem cell (ESC) or pluripotent stem cells (PSC) with dedicated neural lineages [18]. They recapitulate the developmental processes and organization of the human brain and contain functional neurons and astrocytes [19–21]. Moreover, organoids are an excellent model to elucidate the pathogenesis of neurodevelopmental disorders [22–26]. Previous studies demonstrate that human glioma stem cells invade deeply into cerebral organoids and phenocopy human gliomas [27, 28]. This “GLICO” model mimics the development and growth of primary brain tumor in human cortex-like TME and provides a new platform to test the sensitivity of anti-glioma drugs. However, cerebral organoids for non-primary brain tumors (e.g., metastatic brain tumors) have not been evaluated or studied. Therefore, introducing breast cancer cells into cerebral organoids to study BCBM has exciting potential to validate targetable interactions for tumor growth in a new TME.

The crosstalk between human breast cancer cells and TME in brain metastatic process needs more investigation. Using immune-deficient animal models probably insufficiently mimics the cell-cell communication as in human brain cancers [29]. In this study, we generated a novel human-derived breast cancer cell-cerebral organoid co-culture model to elucidate the TME for breast cancer colonization and growth, providing a unique opportunity to study BCBM. We adopted this system to successfully distinguish the metastatic ability of human breast cancer cell lines of MDA-MB-231 and its brain metastatic MDA-MB-231 Br-EGFP line, MCF-7, HCC-1806, and SUM159PT. This new model will provide a valuable platform for molecular, cellular, and genetic approaches to investigate the interaction of breast cancer cells with TME in brain metastasis.

## Results

### Co-culture of human astrocytes with human breast cancer cells

**Fig. 1:**
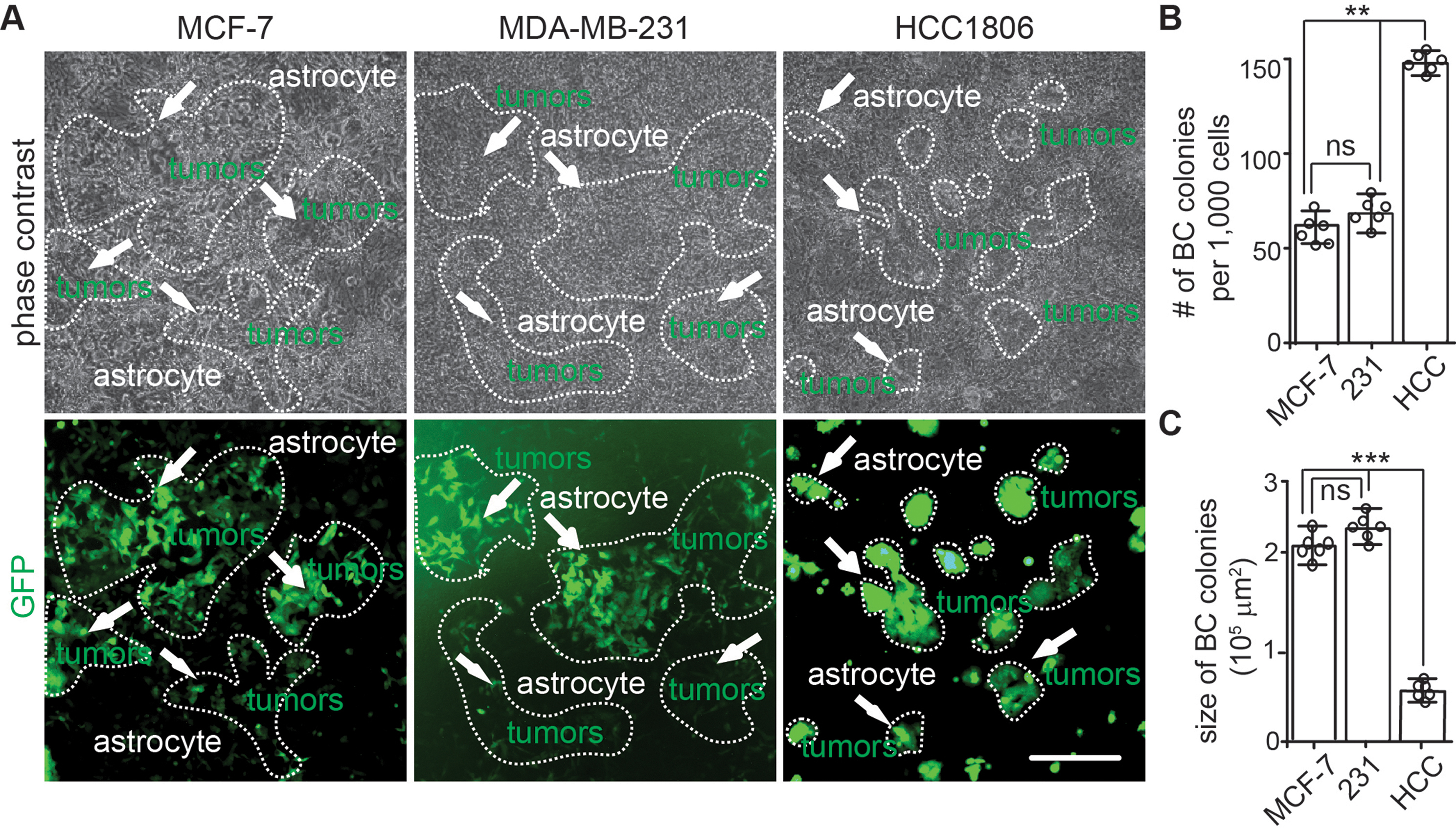
2D co-culture of human breast cancer cells with human astrocytes. (**A**) Phase contrast and GFP fluorescence of MDA-MB-231, MCF-7, and HCC-1806 breast cancer cells on primary human astrocyte monolayer for 10 days. Arrows indicated GFP^+^ breast cancer cell colonies. Dashed lines circled the borders of breast cancer colonies based on GFP expression. (**B**) Mean *±* SE of the number of breast cancer cell colonies formed from 1,000 cells seeded on human astrocytes. (**C**) Mean *±* SE of the size of breast cancer cell colonies cultured on human astrocytes. One-way ANOVA was used for statistical analysis. *n* = 6 independent experiments. ns: no significance; **: *p* < 0.01, ***: *p* < 0.001. Bar = 100 μm

We selected human breast cancer cell lines of MDA-MB-231 [30], MCF-7 [31], and HCC-1806 [32–34] to co-culture with human astrocytes. We chose astrocytes as host because astrocytes are the most abundant brain glia cells and they have been shown to facilitate cancer cell survival, growth, proliferate, and migration at different stages of metastatic outgrowth [35–39]. Recombinant lentivirus encoding green fluorescent protein (GFP) was used to label breast cancer cells for visualization of their growth in co-culture. We seeded 1 × 10^3^/mL single GFP^+^ breast cancer cells onto monolayer human astrocyte in a 35 mm dish. After 10 days, we found that MDA-MB-231, MCF-7, and HCC-1806 cancer cells all formed colonies on human astrocytes (Fig. 1A). The breast cancer cell colonies were not easily identified from human astrocytes under phase contrast microscopy. With GFP fluorescence, we could find MDA-MB-231 and MCF-7 colonies with irregular shapes mixed with human astrocytes (arrows in Fig. 1A). In contrast, the colonies from HCC-1806 cells were round and their individual colonies were separated on the monolayer of human astrocytes (arrows in Fig. 1A). We found relatively higher GFP fluorescent intensity in HCC-1806 colonies, which might be caused by the clustered cells in the colony. The number of breast cancer colonies was about 60 from 1,000 seeded MDA-MB-231 cells and MCF-7 cells (Fig. 1B). Nevertheless, the number of HCC-1806 colonies on astrocytes was significantly higher than the other two breast cancer cell lines (Fig. 1B). Both MDA-MB-231 cells and MCF-7 cells formed larger colonies than HCC-1806 cells (Fig. 1A and C). These results suggested that the human astrocyte-breast cancer cells co-culture model could provide some information for the invasiveness and growth of human breast cancer cells, however, this co-culture system failed to distinguish their potential for colonization in brain TME. It is possible that the monolayer human astrocytes lack other critical cell components and/or extracellular matrix (ECM) to restrict breast cancer cell’s growth as in human brain. This experiment emphasized the development of a better model to study BCBM in culture.

### Mimicking BCBM using hESC-derived cerebral organoids and human breast cancer cells

**Fig. 2:**
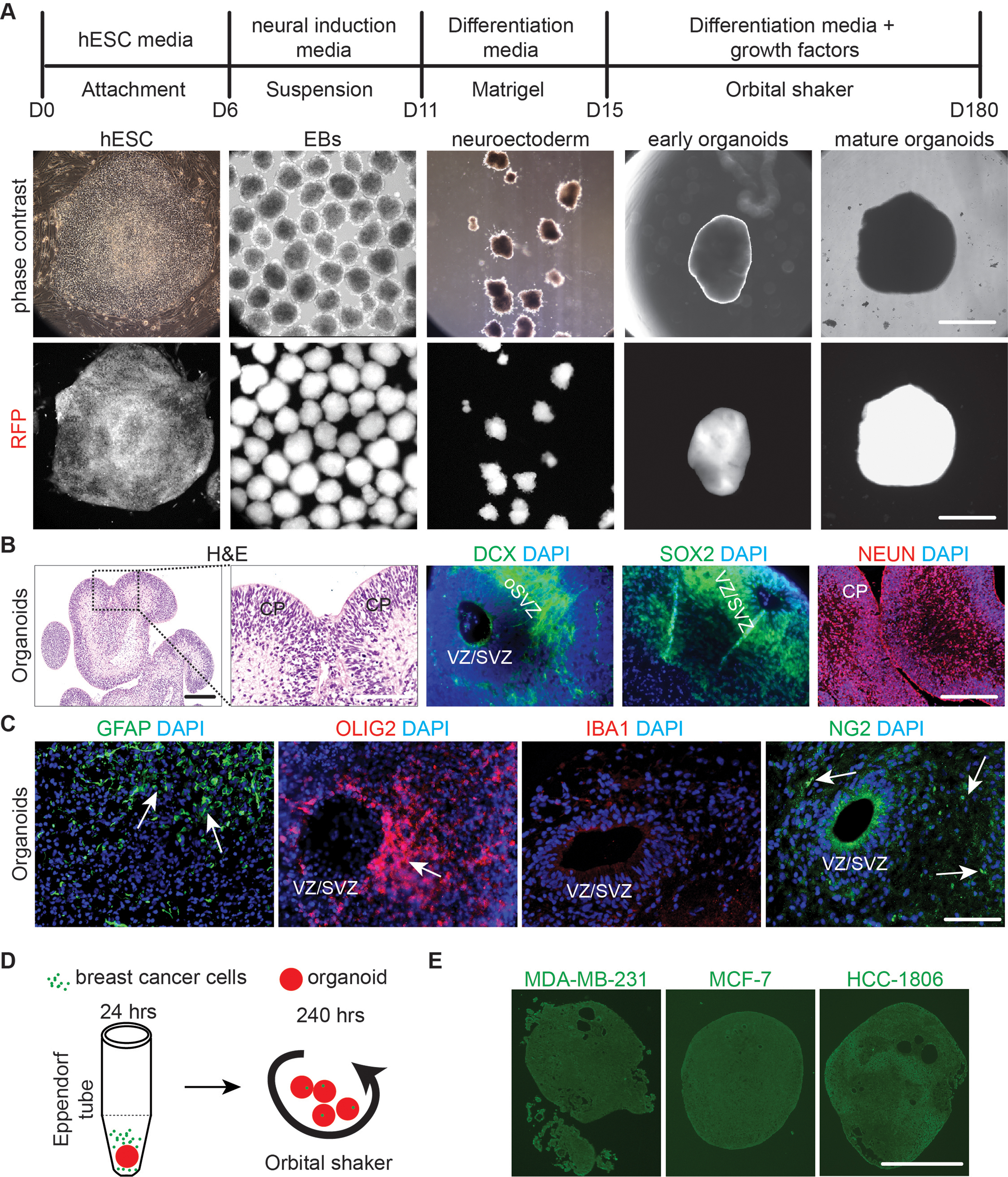
Generation of cerebral organoids and their co-culture with breast cancer cells in Eppendorf tube. (**A**) The schematic flow for generation of cerebral organoids from hESC. The phase contrast and RFP fluorescent images of H9-RFP derived hESC colony, EBs, and organoids at different developmental stages were shown. (**B**) H&E staining and immunofluorescent (IF) staining of DCX, SOX2, NEUN, and DAPI for mature organoids from H9 hESC. (**C**) IF staining of GFAP, OLIG2, IBA1, NG2, and DAPI for mature organoids from H9 hESC. Arrows indicated GFAP^+^, OLIG2^+^ and NG2^+^ cells. (**D**) A schematic depiction for generating organoid-breast cancer cells co-culture using Eppendorf tube. (**E**) GFP fluorescence of MCF-7 cells, MDA-MB-231cells, and HCC-1806 cells co-cultured with H9 organoids in Eppendorf tube. Representative images were from more than 10 cerebral organoids in 2–3 independent experiments. VZ/SVZ: ventricular zone/subventricular zone, CP: cortical plate. Bar = 500 μm in A and E, 100 μm in B and C

Since BCBM occurs frequently in cerebral cortex [40], we propose to generate a new 3D co-culture model to use hESC-derived cerebral organoids as a host for breast cancer cells. The cerebral organoids could mimic the cytoarchitecture of human cerebral cortex and have been widely used to study human brain development and abnormalities [41]. We prepared cerebral organoids with a female hESC line H9 expressing red fluorescent protein (RFP) (designated as H9-RFP). The H9-RFP cells were harvested for generation of embryonic body, immature organoids, and mature cerebral organoids within a time frame of 180 days (Fig. 2A). After we sectioned the mature cerebral organoids, H&E staining indicated the structures of ventricular zone (VZ)/subventricular zone (SVZ)-like regions and cortical plate (CP) (Fig. 2B). For the cell categories in cerebral organoid, we identified doublecortin (DCX) positive immature neurons (9.5 *±* 3.7%) in outer SVZ, Sex Determining Region Y box-2 (SOX2) positive neural progenitors (7.3 *±* 2.5%) in VZ/SVZ, and Neuronal Nuclei (NEUN) positive mature neurons (77.5 *±* 9.3%) in CP of the cerebral organoids (Fig. 2B). We also found glial fibrillary acidic protein (GFAP) positive astrocytes (5.2 *±* 2.7%) and oligodendrocyte transcription factor 2 (OLIG2) positive oligodendrocytic cells (1.2 *±* 0.3%), but not (0%) Ionized calcium binding adaptor molecule 1 (IBA1) positive microglia in these organoids (Fig. 2C). There were minimal percentage of cells (< 0.2%) expressing pericyte marker of chondroitin sulfate proteoglycan 4 (NG2) in the organoids (Fig. 2C). These results indicated neuronal and glial differentiation in cerebral organoids. After confirming these neural characteristics, we transferred single organoid to Eppendorf tubes to incubate with GFP labelled MDA-MB-231, MCF-7, and HCC-1806 cells. The organoids were incubated with breast cancer cells at 1,000/tube or 10,000/tube for 24 h before transferred to 6-well plate on orbital shaker for another 10 days (Fig. 2D). However, we did not observe invasion and growth of these three types of breast cancer cells in organoids by the end of experiment (Fig. 2E; Table 1), suggesting that further optimization was required for this new model.

**Table 1:**
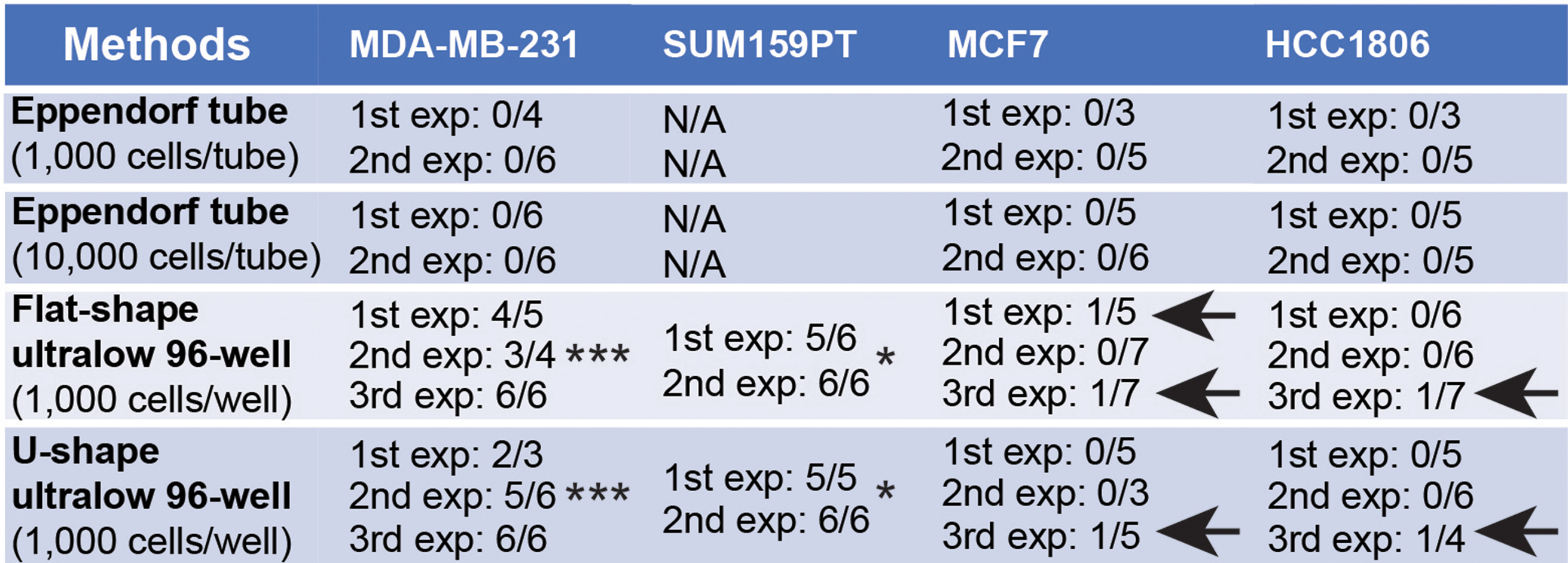
The successful rates for generating cerebral organoid-breast cancer cell co-culture with different methods. Arrows indicated the experiments with growth of MCF-7 and HCC-1806 breast cancer cells in organoids. *: *p* < 0.05 for comparison between SUM159PT with MCF-7 and HCC-1806 breast cancer cells; ***: *p* < 0.001 for comparison between MDA-MB-231 with MCF-7 and HCC-1806 breast cancer cells

### Optimization of breast cancer cell-organoid co-culture model

**Fig. 3:**
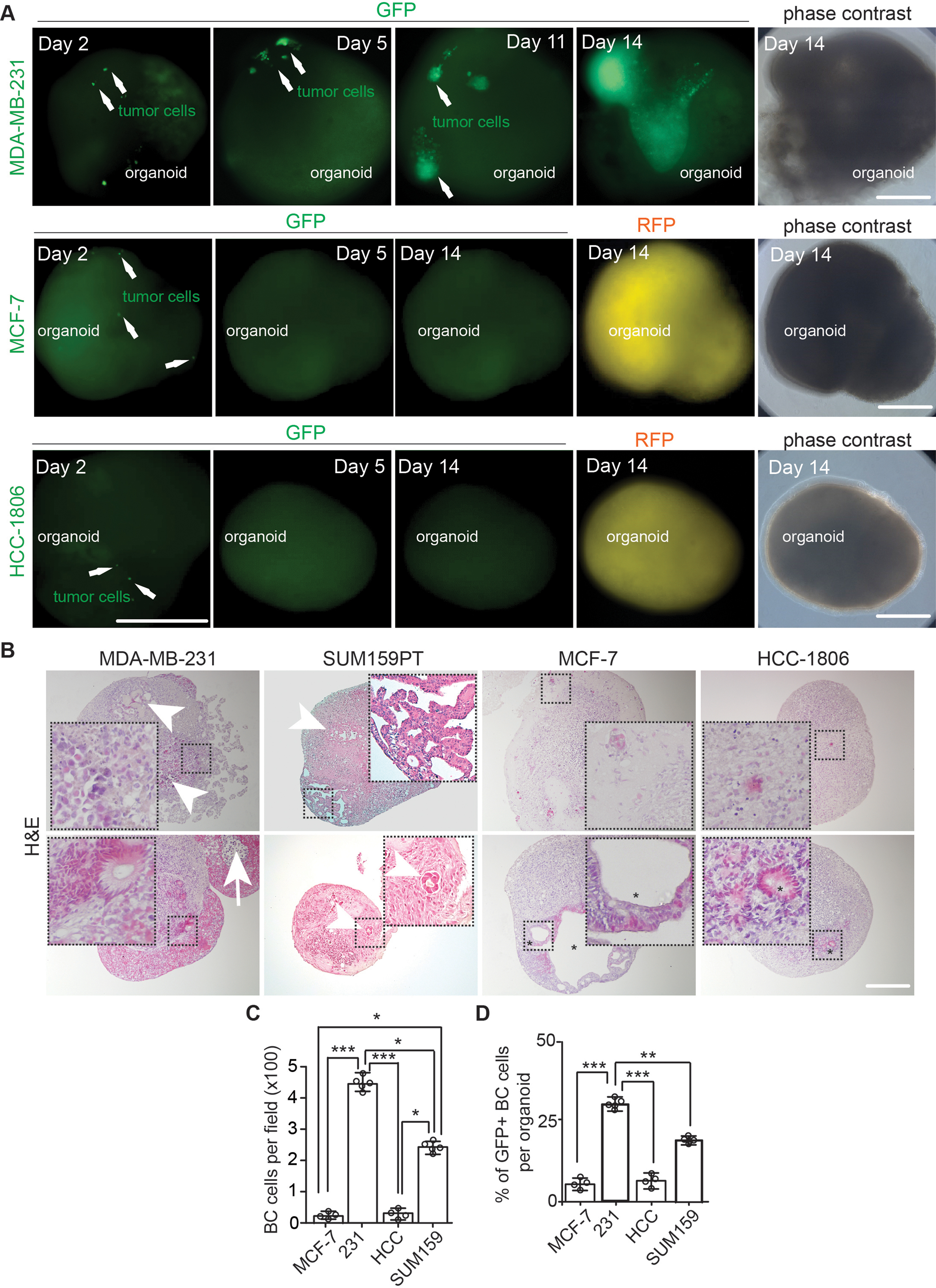
Co-culture of human breast cancer cells with hESC-derived cerebral organoids allowed growth and colonization of MDA-MB-231 and SUM159PT cell lines. (**A**) Phase contrast, GFP fluorescent, and RFP fluorescent images of co-cultured breast cancer cell lines in cerebral organoids from day 2 to day 14. Arrows indicated GFP^+^ breast cancer cells. (**B**) H&E staining of MCF-7 cells, MDA-MB-231 cells, SUM159PT cells, and HCC-1806 cells co-cultured with organoids. The arrow indicated apoptotic MDA-MB-231 cells and arrowheads indicated breast cancer cell colonies in organoids. Stars marked visible colonies from MCF-7 cells and HCC-1806 cells. (**C** and **D**) Mean *±* SE of the number (**C**) of breast cancer cells and the percentage (D) of GFP^+^ breast cancer cells in organoid-breast cancer co-cultures. Representative images and quantification were from 13–19 cerebral organoids in 4–5 independent experiments. One-way ANOVA was used for statistical analysis. *: *p* < 0.05, **: *p* < 0.01, ***: *p* < 0.001. Bar = 200 μm

To minimize the potential impacts of Eppendorf tube on co-culture, we transferred a single cerebral organoid to one well of a 96-well Ultralow attachment plate (both U shape bottom and flat bottom plates were used in the subsequent experiments) and seeded 1,000 breast cancer cells for each organoid. One day later (D2), we transferred the organoids back to the 6-well plate on an orbital shaker for continuous culturing. We found invasion of single MDA-MB-231, MCF-7, and HCC-1806 breast cancer cells into organoids (Fig. 3A, arrows). Our continuous fluorescent recording identified 2–3 small colonies of GFP^+^ MDA-MB-231 breast cancer cells in organoid at D5 (Fig. 3A, arrows). These MDA-MB-231 tumor colonies in cerebral organoids robustly expanded and fused into bigger colonies from D11 to D14 (Fig. 3A). We found more than 85% of organoids co-cultured with MDA-MB-231 cells contained GFP^+^ tumor colonies (Table 1). We also used another metastatic TNBC of SUM159PT cells [42, 43] to co-culture with cerebral organoids and our results indicated that 96% of co-cultured organoids showed tumor colonies after 14 days incubation (Table 1). In contrast, we did not find obvious MCF-7 and HCC-1806 breast cancer colonies in cerebral organoids at D5 (Fig. 3A). At D14, more than 90% of organoids co-cultured with MCF-7 cells and more than 94% of organoids co-cultured with HCC-1806 cells failed to generate visible breast cancer colonies (Fig. 3A; Table 1). Only several MCF-7 and HCC-1806 breast cancer cell-organoid co-cultures showed tumor colonies (arrows in Table 1).

Next, we sectioned the organoids co-cultured with MDA-MB-231, SUM159PT, MCF-7, and HCC-1806 cells at D14. H&E staining indicated that MDA-MB-231 cells formed multiple large tumor colonies in (Fig. 3B, arrowheads in top panel) or on the surface of cerebral organoids (Fig. 3B, bottom panel). The growth of MDA-MB-231 tumor colony could result in apoptosis in its center (Fig. 3B, arrow in bottom panel). The breast cancer colonies on organoid surface not only protruded outside but also penetrated the organoids (Fig. 3B, inset in bottom panel). We observed the formation of tumor colonies with lumen-like structures in SUM159PT cell-cerebral organoid co-cultures (Fig. 3B). Most of MCF-7- organoid and HCC-1806-organoid co-cultures did not exhibit obvious tumor colonies. We observed some “mini” clusters composed of several breast cancer cells in these organoids (Fig. 3B, insets in top panels). Only under some exceptional circumstances for MCF-7 and HCC-1806 cells (selected samples indicated by arrows in Table 1), we could find tumor colonies in organoids (Fig. 3B, asterisk in bottom panels). We counted the cancer cell number in co-cultured organoids, and we found significantly more MDA-MB-231 cells and to a less extent, SUM159PT cells than MCF-7 cells and HCC-1806 cells (Fig. 3C). Our analysis of the GFP^+^ breast cancer cells in organoids obtained similar results as what we found by H&E staining (Fig. 3D). Taken together, these results suggested that human cerebral organoids allowed the growth of some types of breast cancer cell lines for colony formation.

### Characterization of co-cultured breast cancer cells and cerebral organoids

**Fig. 4:**
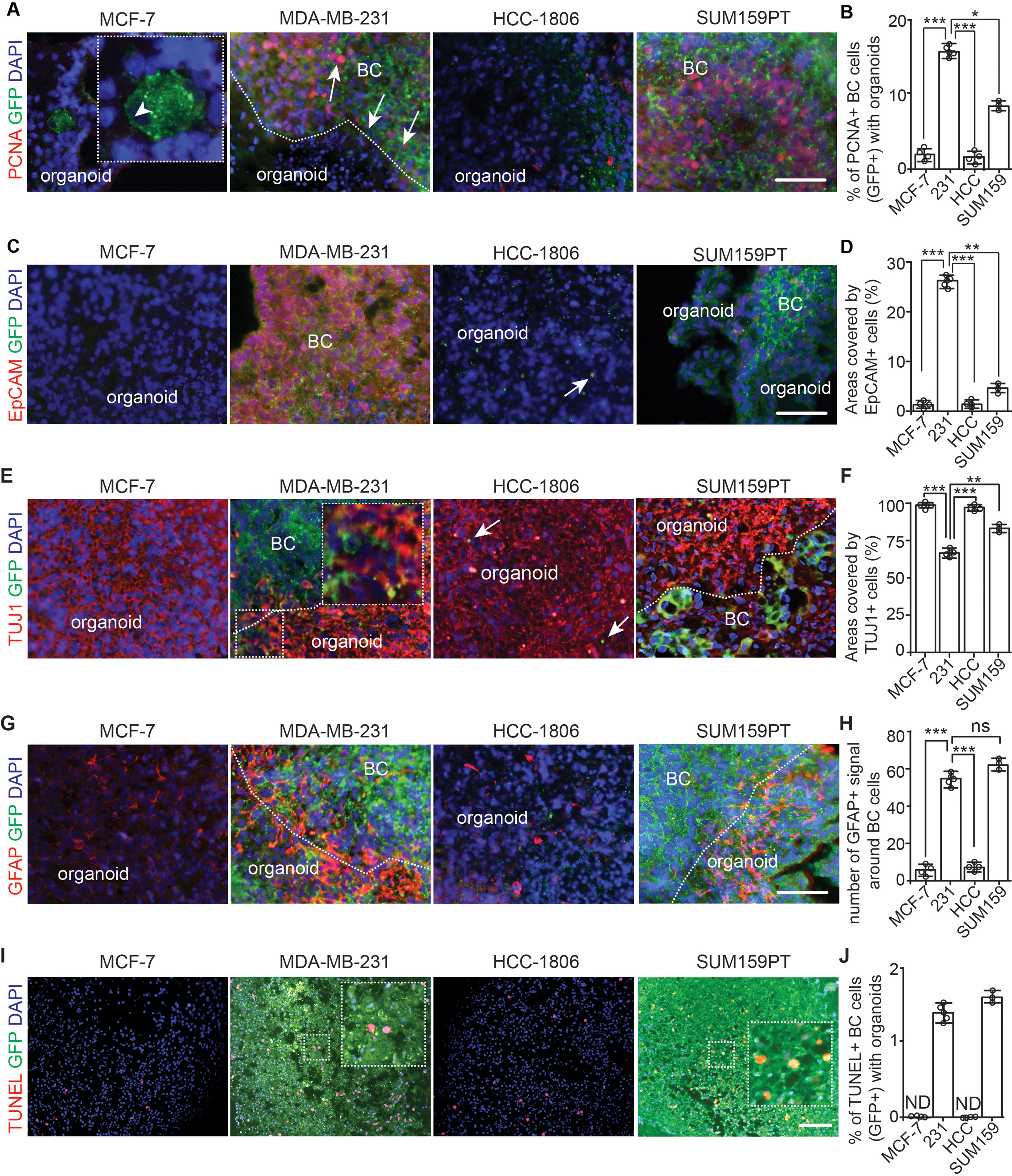
Growth of breast cancer cells in cerebral organoids. (**A**) IF of PCNA and GFP of breast cancer cells co-cultured with organoids at day 14. (**B**) Mean *±* SE of the percentage of PCNA^+^ proliferative GFP^+^ breast cancer cells in cerebral organoids. (**C**) IF of EpCAM and GFP of breast cancer cells co-cultured with organoids. The arrow indicated a dead GFP-HCC-1806 cells in cerebral organoid. (**D**) Mean *±* SE of the areas occupied by EpCAM^+^ breast cancer cells in cerebral organoids. (**E**) IF of TUJ1 and GFP of breast cancer cells co-cultured with organoids. Boxed area shown in detail as inset. Arrows indicated apoptotic HCC-1806 cells. (**F**) Mean *±* SE of the percentage of the areas occupied by TUJ1^+^ cells in co-cultured cerebral organoids. (**G**) IF of GFAP and GFP of breast cancer cells co-cultured with organoids. (**H**) Mean *±* SE of the number of GFAP^+^ cells in cerebral organoids around tumor colonies. (**I**) Fluorescence of TUNEL and DAPI of co-cultured breast cancer cells with organoids. Boxed area shown in detail as inset. (**J**) Mean *±* SE of the percentage of TUNEL^+^ apoptotic breast cancer cells co-cultured with cerebral organoids. Dashed line indicated the border of breast cancer cells within organoid. Selected areas were shown in detail as inset. Representative images and quantification were from 13–19 cerebral organoids in 4–5 independent experiments. One-way ANOVA was used for statistical analysis. ND: not determined, ns: no significance, *: *p* < 0.05, **: *p* < 0.01, ***: *p* < 0.001. Bar = 100 μm

We analyzed the proliferation of breast cancer cells in cerebral organoids by immunostaining of proliferative cell nuclei antigen (PCNA) at D14. We found that ~ 15% of GFP^+^ MDA-MB-231 and ~ 10% of GFP^+^ SUM159PT breast cancer cells were PCNA^+^ in the cerebral organoids (Fig. 4A and B). The GFP^+^ PCNA^+^ cells from co-cultured MCF-7 and HCC-1806 cells were significantly fewer (Fig. 4A and B). MDA-MB-231 cells formed GFP^+^ EpCAM^+^ epithelial colonies, which occupied more than 20% of the area of sectioned cerebral organoids (Fig. 4C and D). However, we did not find obvious EpCAM expression in co-cultured MCF-7, HCC-1806, and SUM159PT breast cancer cells (Fig. 4C and D). We noticed the invasiveness of MDA-MB-231 and SUM159PT tumor colonies in the cerebral organoids to occupy beta tubulin-3 (TUJ1) positive neuronal areas (Fig. 4E). In most MCF-7 cells and HCC-1806 cells co-cultured organoids, we hardly found clusters of GFP^+^ tumor cells and occasionally, we observed some condensed GFP^+^ HCC-1806 cell bodies surrounded by TUJ1^+^ processes (Fig. 4E and F). Interestingly, the GFAP staining indicated that there was a robust increase of GFAP intensity around MDA-MB-231 cells and SUM159PT cells in organoids (Fig. 4G). We did not find GFP^+^ MCF-7 and GFP^+^ HCC-1806 cancer cells around GFAP^+^ cells in cerebral organoids (Fig. 4G). The number of GFAP^+^ signals in organoids co-cultured with MDA-MB-231 cells and SUM159PT cells was significantly higher than that in organoids co-cultured with MCF-7 cells and HCC-1806 cells (Fig. 4H). We used TUNEL to examine the apoptosis of GFP^+^ breast cancer cells in organoids. Approximately 1.3% of MDA-MB-231 cells and SUM159PT cells underwent apoptosis (Fig. 4I and J). The incidence of apoptotic MCF-7 and HCC-1806 cells was undetectable because of their low number in the co-culture (Fig. 4I and J). Taken together, our data confirmed that MDA-MB-231 cells and SUM159PT cells proliferate to colonize TUJ1^+^ regions and increased GFAP signals in invaded cerebral organoids.

### Comparison of MDA-MB-231 and its brain prone derivative in cerebral organoids

**Fig. 5:**
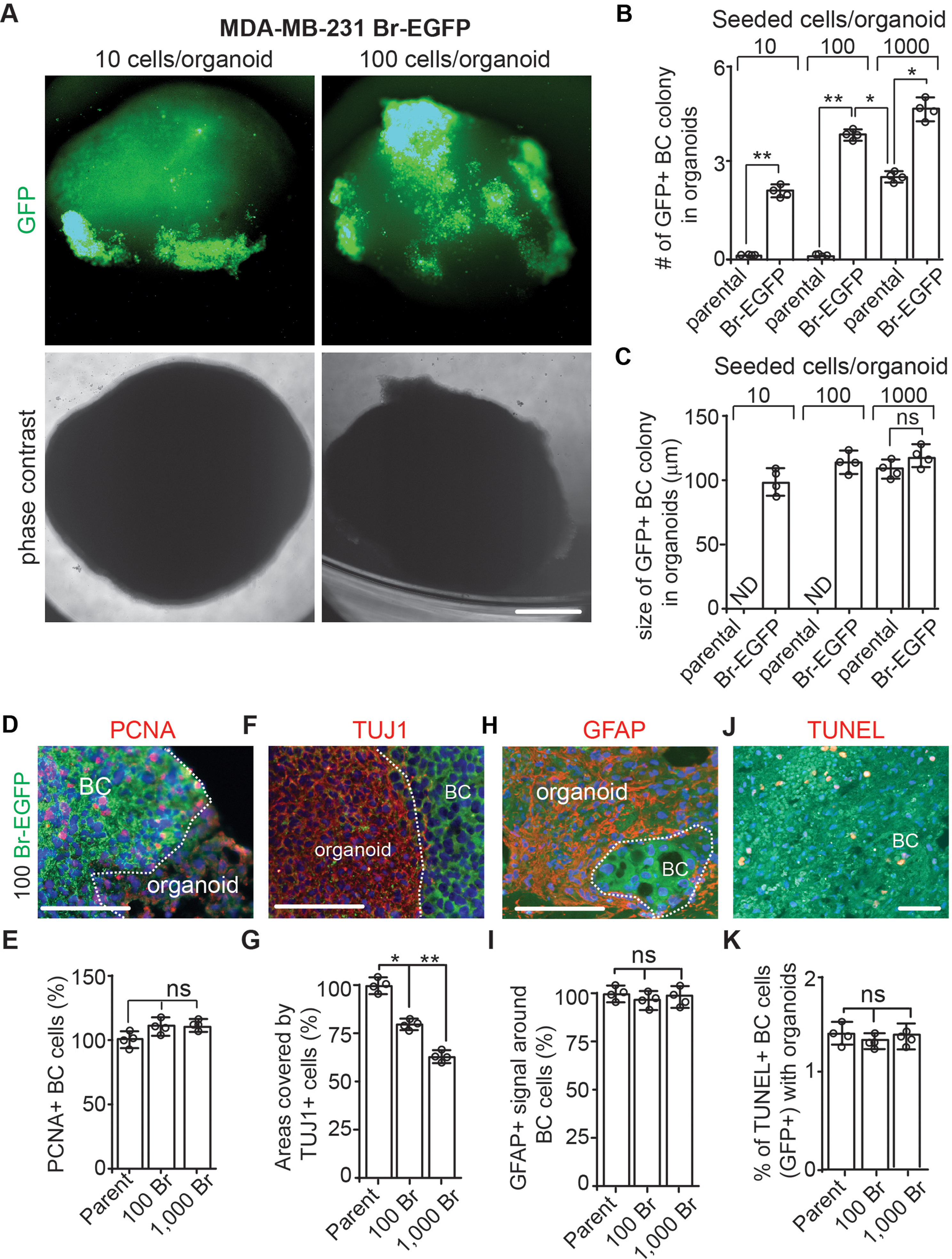
Increased tumor colony formation in cerebral organoid from MDA-MB-231 Br-EGFP cells. (**A**) Phase contrast and GFP fluorescence of co-cultured MDA-MB-231 Br-EGFP breast cancer cells in cerebral organoids at 10 cells/organoid and 100 cells/organoid for 14 days. (**B**) Mean *±* SE of the number of GFP^+^ tumor colonies from parental MDA-MB-231 and MDA-MB-231 Br-EGFP cells in cerebral organoids. (**C**) Mean *±* SE of the size of GFP^+^ tumor colonies from parental MDA-MB-231 and MDA-MB-231 Br-EGFP cells in cerebral organoids. (**D**, **F**, **H**, and **J**) IF of PCNA (**D**), TUJ1 (**F**), GFAP (**H**), TUNEL (**J**) with GFP and DAPI of Br-EGFP breast cancer cells co-cultured with cerebral organoids. (**E**, **G**, **I**, **K**) Mean *±* SE of the relative percentage of PCNA^+^ cells (**E**), TUJ1^+^ areas (**G**), number of GFAP^+^ signaling around tumor colonies (**I**), and TUNEL^+^ apoptotic breast cancer cells (**K**) from parental MDA-MB-231 (set as 100%) and 100-1,000 MDA-MB-231 Br-EGFP cells in cerebral organoids. Representative images and quantification were from 12 cerebral organoids in 4 independent experiments. Two-way ANOVA and Student’s t-test were used for statistical analysis. ND: not determined, ns: no difference, *: *p* < 0.05, **: *p* < 0.01. Bar = 200 μm for A, 100 μm for D, F, H, and J

To confirm the potential of breast cancer cell-cerebral organoid co-culture to distinguish the cancer cell’s brain metastatic ability, we compare the behaviors of parental MDA-MB-231 and MDA-MB-231 Br-EGFP (Br-EGFP in short) with increased brain metastatic capacity [44] in organoids. We seeded parental MDA-MB-231 cells and Br-EGFP cells at 10 cells, 100 cells, and 1,000 cells per organoid. We found that parental GFP-231 cells could not form tumor colony at concentrations of 10 cells and 100 cells per organoid while Br-EGFP cells exhibited multiple colonies in organoids at the same concentrations (Fig. 5A; Table 2). The number of Br-EGFP tumor colonies averaged 2 per organoid at 10 cells and 4 per organoid at 100 cells (Fig. 5A and B). Even though parental MDA-MB-231 cells formed multiple colonies at 1,000 cells, the number was fewer than those from Br-EGFP cells at 100 and 1,000 cells per organoid (Fig. 5B). We compared the size of GFP^+^ colonies and we did not notice significant difference between tumor colonies from 1,000 parental GFP-231 cells and Br-EGFP cells at different concentrations (Fig. 5C), suggesting comparable growth rates once these cancer cells colonized the organoids. Next, we performed PCNA staining, and we found a similar number of PCNA^+^ cells between parental GFP-231 cells and Br-EGFP cells in organoids (Fig. 5D and E). With more Br-EGFP tumor colonies formed in organoids, the TUJ1^+^ areas decreased (Fig. 5F and G). The GFAP staining and TUNEL staining did not show significant difference between parental GFP-231 cells and Br-EGFP cells in organoids (Fig. 5H, I, J and K). These data suggested the feasibility of using cerebral organoids to compare breast cancer cells’ brain metastatic potential.

**Table 2:**
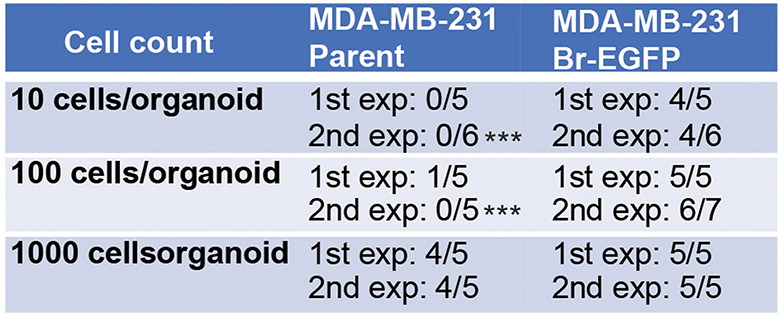
Serial dilution of MDA-MB-231 parental cells and MDA-MB-231 Br-EGFP cells to co-culture with cerebral organoids. Statistical differences between groups for limiting dilution were performed using ELDA. ***: *p* < 0.001

### Investigating breast cancer cell-TME cell interaction in cerebral organoids using sLP–mCherry proximate labeling system

**Fig. 6:**
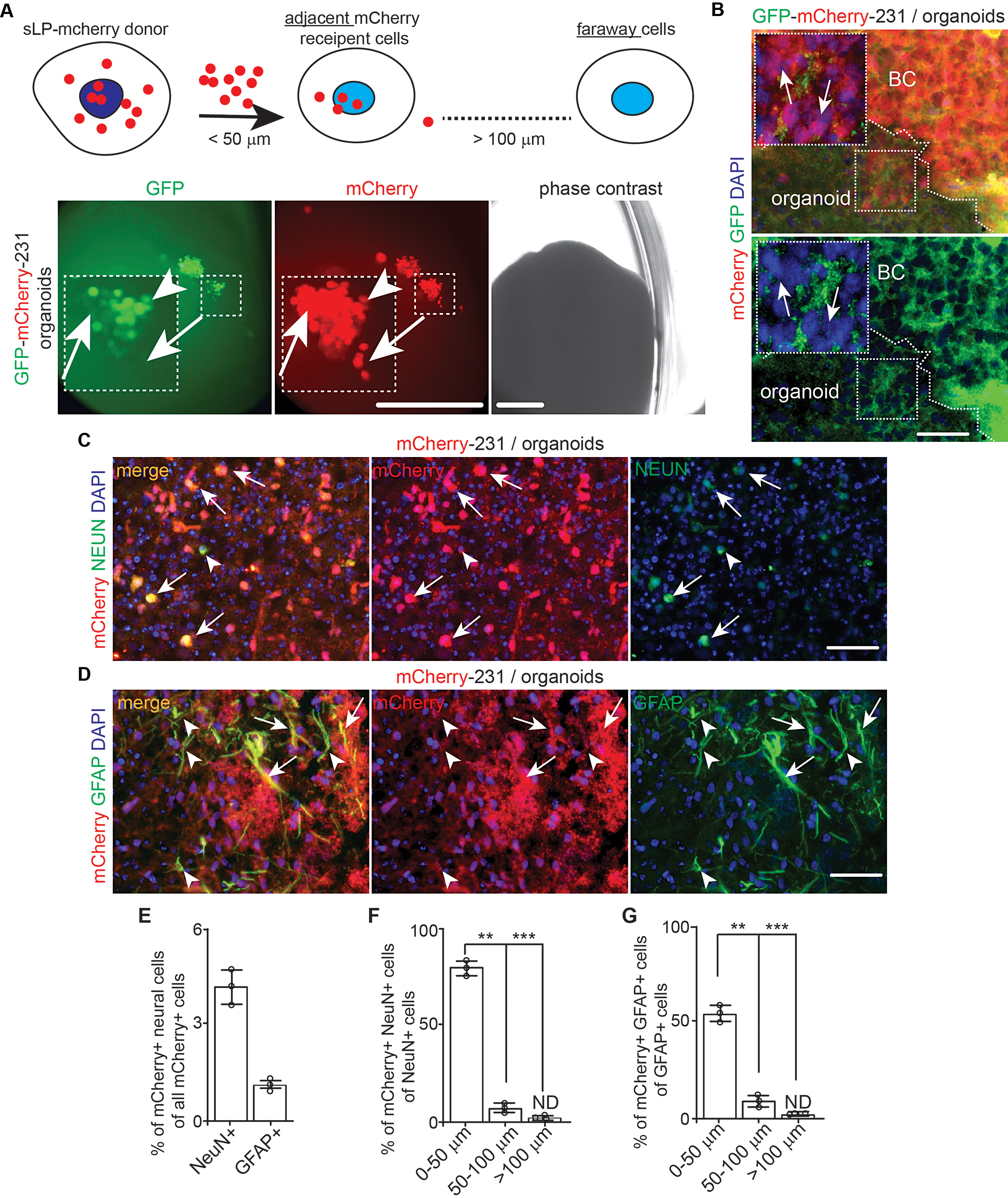
The transfer of sLP–mCherry from MDA-MB-231 breast cancer cells to neurons and astrocytes in cerebral organoids. (**A**) A schematic description for sLP-mCherry system to label neighbor recipient cells. Phase contrast and fluorescent images of GFP and mCherry of MDA-MB-231 cells in naïve WIBR3 organoids for 7 days. Arrows indicated mCherry^+^ organoid cells. Arrowhead indicated mCherry^+^ GFP^+^ breast cancer cells. Boxed area shown in detail as inset. (**B**) Fluorescence of GFP, mCherry, and DAPI of MDA-MB-231 cells in WIBR3 derived cerebral organoids. Arrows indicated mCherry^+^ cells in organoids. The dotted line indicated the boundary of breast cancer cells and organoids. Boxes shown in detail as inset. (**C**) IF of NEUN, mCherry, and DAPI of cerebral organoid cells and MDA-MB-231 cells expressing sLP–mCherry. Arrows indicated mCherry^+^ neurons while arrowheads indicated mCherry^−^ neurons. (**D**) IF of GFAP, mCherry, and DAPI of cerebral organoid cells and MDA-MB-231 cells expressing sLP–mCherry. Arrows indicated mCherry^+^ astrocytes while arrowheads indicated mCherry^−^ astrocytes. (**E**) Mean *±* SE of percentage of NEUN^+^ mCherry^+^ cells and GFAP^+^ mCherry^+^ cells of total mCherry^+^ cells in the organoids. (**F** and **G**) Mean *±* SE of percentage of NEUN^+^ mCherry^+^ cells of total NeuN^+^ cells (**F**) and GFAP^+^ mCherry^+^ cells of total GFAP^+^ cells (**G**) within different radius to tumor colony in organoid. Representative images and quantification were from 10 cerebral organoids in 3 independent experiments. One-way ANOVA and Student’s t-test were used for statistical analysis. ND: not determined, **: *p* < 0.01, ***: *p* < 0.001. Bar = 200 μm for A, 50 μm for B, C, and D

We adopted a previously described sLP–mCherry system [45] to identify the direct contact of breast cancer cells with neighbor cells in organoid (Fig. 6A). We engineered MDA-MB-231 cells to stably express both sLP–mCherry and GFP (designated as GFP-mCherry-231) or sLP–mCherry alone (designated as mCherry-231). We first used GFP-mCherry-231 cells to co-culture with cerebral organoids from WIBR3 hESC (another independent line from a female donor [46]). Seven days later, at the borders of GFP^+^ mCherry^+^ tumor colonies (Fig. 6A, arrowhead) and organoids, we identified single or clustered mCherry^+^ organoid cells (Fig. 6A, arrows). In the sectioned co-culture, we found single mCherry^+^ organoid cells (Fig. 6B, arrows). To identify the neural cells in co-cultured organoids, we used mCherry-231 cells, and we double stained mCherry with neural markers at D14. We found that 4% of total mCherry^+^ cells were NEUN^+^ neurons in the co-cultured organoids (Fig. 6C and E). GFAP staining indicated that 1.5% of total mCherry^+^ cells in organoids were GFAP^+^ astrocytes (Fig. 6D and E). We found that the majority of mCherry^+^ neural cells were within 50 μm distance to mCherry-231 tumor cells (Fig. 6F and G). These results indicated that both neurons and astrocytes in organoids could be labelled with sLP-mCherry secreted from neighbor breast cancer cells.

## Discussion

With the development of technologies for early detection, advances in surgery, radiotherapy and chemotherapies, breast cancer patients without distal metastasis have a high survival rate of 91% [47]. However, this number is very low once breast cancer cells metastasize to the brain. Overall, the average survival rates for BCBM patients without treatment range from 2** to **25 months [48]. One of the reasons is that BCBM research is far lagging primary breast cancer research. In practice, there are limited BCBM models for basic researchers. Additionally, the human brain TME is critical for BCBM, and it has become increasingly evident that the BCBM is only possible with a permissive TME [49].

To overcome the difficulty for lacking human relevance, we generated a co-culture system employing hESC-derived cerebral organoids and human breast cancer cell lines to establish a novel BCBM model. Using this model, we characterized several key features of human BCBM in culture. First, cerebral organoids distinguished the ability of human breast cancer cell lines to form tumor colonies in a new TME, which suggested the future application of this platform to reduce transplantation of human breast cancer cells with uncertain brain tropism. Second, this system provided an excellent opportunity for the time-lapse recording to trace breast cancer cell invasion, colony formation, and fusion of colonies in culture. We noticed the expression of EpCAM in mesenchymal like MDA-MB-231 cells in organoids (Fig. 4E and F). This raised interesting possibilities that MDA-MB-231 cells with higher expression level of EpCAM survived in organoids or they performed mesenchymal-epithelia transition in organoids to re-express EpCAM. Third, we detected the degeneration of TUJ1^+^ neurons and the astroglial response with increased GFAP intensity to brain metastasis in cerebral organoids (Fig. 4E and H). Loss of neuron is a marker of neurodegeneration by brain tumors and the excessive glutamate release from non-neuronal cells is considered as a mechanism for neuronal death [50, 51]. The astrocytes are hijacked by brain tumor cells to reactive with proliferation, hypertrophy, and enhanced expression of GFAP [52, 53]. The higher GFAP intensity in MDA-MB-231 cells and SUM159PT cells (Fig. 4G and H) co-cultured organoid suggested that these breast cancer cells induced a higher expression of GFAP, or they enhanced the transition of progenitors to GFAP^+^ astrocytes. Taken together, our co-culture model could mimic BCBM from both aspects of neural cells and breast cancer cells, which will facilitate our study of their interactions.

The inoculation of sLP-mCherry-231 breast cancer cells allowed us to determine their interaction with various cell types in cerebral organoid. We found that a small population of the total stromal cells were labeled with secreted mCherry from breast cancer cells (Fig. 6). We also found that more NEUN^+^ neurons than GFAP^+^ astrocytes in the co-cultured cerebral organoids were labelled with mCherry from sLP-mCherry-231 cancer cells (Fig. 6). It is possible that the mCherry released from breast cancer cells could be delivered retrogradely from the TUJ1^+^ process to the center cell body of these neurons. It is known that astrocytes had a smaller cell body and shorter processes, which may reduce their chances of receiving mCherry signals from breast cancer cells. Irrespective of the difference in labeling for neurons and astrocytes, the sLP-mCherry system will be useful to study the cell-cell interaction in organoids in future research.

The 3D cerebral organoid-breast cancer cell co-culture showed superiority to 2D co-culture system (Fig. 1) to distinguish the brain tropism of human breast cancer cells for colonization. The differences between astrocytes and cerebral organoid might be resulted from the more complicated cell types, their functions, as well as ECM compositions and stiffness in 3D culture [54–56]. The cerebral organoids contain both mature and immature neurons, progenitors, and glia [57, 58]. It is revealed that the neuronal activities stimulate the growth and invasion of primary and metastatic brain tumors in vivo [59–63]. It is reported that metastatic breast cancer cells receive and respond to neuronal signals [63] that might facilitate their growth in organoids. The ECM in 3D cerebral organoids is more complicated, which has been proved to be an advantage for modeling neurodegenerative brain disorders [64]. Previous results demonstrate that increasing the dimensionality of ECM around cells can drastically impact cell proliferation, differentiation, mechano-responses, and cell survival [65–67]. These features can be achieved in cerebral organoid but not on monolayer astrocytes. Together, the 3D co-culture model could provide a tool to mimic BCBM from human breast cancer cells with brain tropism.

Previous studies have used patient derived glioma for the GLICO model to study the invasion of glioma cells in cerebral organoids [27]. It needs to be emphasized that gliomas originate from CNS cells [68, 69]. It is reasonable to expect that most, if not all glioma cells, could re-establish a tumor colony in brain TME. Nevertheless, breast cancer cells are from mammary gland with different organotropism for metastasis [70, 71], ranging from 10 to 16% for different subtypes. Except for a few established human breast cancer cell lines that can be used to study brain metastasis in vivo, the information for brain tropism of human breast cancer patient derived xenograft model (PDX) and patient derived organoid (PDO) model remains limited [17, 72]. A previous study uses only MDA-MB-231 cells to co-culture with organoid and examines the epithelia to mesenchymal transition of breast cancer cells [73]. Nevertheless, this study does not compare with brain metastatic subclones of MDA-MB-231 cells or with other breast cancer cell lines to colonize organoids and limits the application of their co-culture system. Since the BCBM research field still lacks cost efficient experimental systems to establish brain tropism model from PDX or PDO, it will be plausible to use our cerebral organoid-breast cancer cell co-culture model to accelerate the generation of PDO and PDX BCBM models.

Even though using cerebral organoids had advantages for studies of human BCBM, this model has several limitations in term of convenience, expense, and others [55]. The generation of cerebral organoids is more expensive, and it will take much longer time than monolayer cell culture for differentiation of neural cell types [74]. Moreover, it is appreciated that using hESC to model cerebral organoids needs different technical skills, materials, and equipment, all of which require longer training period for reliable cerebral organoids. For cerebral organoid itself, it is obvious that this model lacks other major cell components of the brain, such as microglia and endothelia-vascular system. Future studies are aimed at overcoming these disadvantages. For example, both microglia and endothelial cells can be reconstituted into brain organoids to partially restore the innate immune and vascular features of brain [75–77]. Our labs also developed a model to mimic human blood-brain barrier with hPSC [78]. These modified models will provide additional strength to study the breast cancer cell extravasation from vascular system and the crosstalk between breast cancer cells with myeloid cells for BCBM.

## Conclusion

Using a new model from hESC-derived brain organoids and multiple human breast cancer cell lines, our results suggest that this new system has the potential to distinguish human breast cancer cell’s metastatic ability in human brain.

## Materials and methods

### Human astrocyte culture

Human astrocytes (HA1800) were purchased from ScienCell (CA). Human astrocytes were primary cultures obtained from human fetal brain tissue. They were isolated and maintained in the presence of 10% fetal bovine serum (FBS)(ThermoFisher Scientific, MA). The cells were plated onto 6-well tissue culture dishes in incubator (37 °C, 5% CO_2_ and 95% humidity). Medium was changed 3 days after plating. Human astrocytes were used for co-culture experiments when they were over 95% confluent. We changed media twice every week and mycoplasma testing were performed to exclude the contamination.

### Culture of breast Cancer cells and co-culture of breast Cancer cells with astrocytes

Human breast cancer cell lines of MCF-7, MDA-MB-231, HCC-1806, and SUM159PT were gifted from Dr. Jun-Lin Guan in Department of Cancer Biology, University of Cincinnati College of Medicine. MDA-MB-231 Br-EGFP cell line was gifted from Dr. Siyuan Zhang in Department of Pathology, University of Texas Southwestern Medical Center [79]. These breast cancer cells were maintained in DMEM-F12 complete media supplemented with 10% FBS, and 1% P/S (Sigma, MO) in a 5% CO_2_ incubator at 37 °C. Media was changed every 2–3 days and cells were passaged when they reached 65–80% confluency.

Breast cancer cells from a 70–80% confluent culture were dissociated by 0.2% trypsin/EDTA solution (ThermoFisher Scientific, MA) for single cell suspension. For co-culture with human astrocytes, 1,000/mL single breast cancer cells in DMEM-F12 with 2% FBS were seeded onto culture. The breast cancer cells were allowed to attach in the human astrocyte monolayer for 24 h and unattached cells will be removed by 3 times wash with DMEM-F12. The attached breast cancer cells grew in 2% FBS DMEM-F12 with media change every 2–3 days. The co-cultured breast cancer cells and astrocytes will be used for analysis 10 days after initial seeding.

### Human embryonic stem cells (hESC) cultures

H9 hESCs were previously described [80]. WIBR3 hESCs were gifted from Dr. Helen Bateup at University of California, Berkeley [24]. hESC culture was carried out as previously described [24]. Briefly, all hESC lines were maintained on a layer of inactivated mouse embryonic fibroblasts (MEFs, CD-1 strain, Charles River) in hESC medium composed of E8 and DMEM/F12 (Thermo Fisher Scientific, MA) supplemented with 20% KnockOut Serum Replacement (KSR) (Thermo Fisher Scientific, MA), 2 mM L-glutamine (Thermo Fisher Scientific, MA), 1% nonessential amino acids (Thermo Fisher Scientific, MA), 0.1 mM 2-mercaptoethanol (Thermo Fisher Scientific, MA), 1% P/S (Sigma, MO), and 4 ng/mL fibroblast growth factor (FGF)-Basic (AA 1-155) recombinant human protein (Thermo Fisher Scientific, MA). Cultures were passaged every 7 days with collagenase type IV (1.5 mg/mL; Thermo Fisher Scientific, MA) and gravitational sedimentation by washing 3 times in wash media composed of DMEM/F12 supplemented with 5% FBS and 1% P/S. All hESC lines were tested monthly and confirmed negative for *Mycoplasma* contamination.

To obtain H9-RFP cells, we utilized the lentiviral vector EF.CMV.RFP from Addgene (#17,619) to generate Lenti-CMV-RFP virus for infecting H9 cells. The ESCs were maintained in E8 medium throughout the experiment. Following viral transduction, we cultured the stem cells for one week and monitored RFP expression using a fluorescent microscope to verify successful transduction. Subsequently, we harvested the transduced ESCs and generated a single-cell suspension using ReLeSR™. Employing fluorescence-activated cell sorting, we isolated RFP-positive cells and seeded them into individual wells of a culture plate to establish single-cell clones. After identifying optimal clones, we expanded their culture for more than two passages while continuously monitoring RFP expression to ensure stable integration. Finally, we confirmed the pluripotency karyotype and stemness of the H9-RFP cells.

### Generation of cerebral organoids from hESC

Generation of forebrain organoids from hESCs was performed as described previously with minor modification [18, 80]. Briefly, hESCs were detached by incubation with Collagenase IV (Thermo Fischer Scientific, MA) for 60 min, transferred to incubate with Accutase (Stemcell Tech, Canada) for 2 min to get single cell suspension. The cell suspension was centrifuged in 800 μm AggreWell plate (Stemcell Tech, Canada) at room temperature with 400 *g* for 1 min and cultured in mTeSR plus media supplemented with 10 µM Y-27,632 (Stemcell Tech, Canada) for 2 days for Embryoid Body (EB) aggregation. On day 3–7, EBs were transferred to an Ultra-low Attachment 6-well plate (Corning Costar, NY) and cultured in H1 neural induction medium containing DMEM/F12 supplemented with 20% KnockOut Serum Replacement, 1% P/S, 1% MEM-NEAAs, 1% GlutaMAX, 0.1 mM 2-mercaptoethanol, 0.0002% heparin, 5 µM SB-431,542 (Stemcell Tech, Canada) and 1 µM LDN-193,189 (Stemcell Tech, Canada). On day 6, half of the medium was replaced with F2 forebrain induction medium containing DMEM/F12 supplemented with 1% N2 supplement, 1% P/S, 1% MEM-NEAAs, 1% GlutaMAX, 0.1 mM 2-mercaptoethanol, 1 µM SB-431,542, and 1 µM CHIR99021 (Stemcell Tech, Canada). On day 7, organoids were embedded in Matrigel (Corning Costar, NY) and cultured in F2 medium for 7 days. On day 14, embedded organoids were dissociated from Matrigel by gentle pipetting, transferred to an ultra-low Attachment 6-well plate placing on a CO_2_ resistant orbital shaker (Thermo Fisher Scientific, MA) and cultured in H3 differentiation medium containing DMEM/F12 supplemented with 1% B27 supplement, 2% N2 supplement, 1% P/S, 1% MEM-NEAAs, 1% GlutaMAX, 0.1 mM 2-mercaptoethanol, and 3 mg/L human insulin (Sigma, MO). The organoids were cultured in H3 media for 1 month and transferred to F4 differentiation medium containing DMEM/F12 supplemented with 1% B27 supplement, 1% P/S, 1% MEM-NEAAs, 1% GlutaMAX, 0.1 mM 2-mercaptoethanol, 20 µg/L GDNF (PeproTech, NJ) and 20 µg/L BDNF (PeproTech, NJ) for 2 months before used for co-cultured experiments.

### Co-culture of breast Cancer cells with Organoids

For co-culture experiments, we used 2 different protocols for colonization of breast cancer cells in organoids. For protocol one, individual organoids were transferred to a 1.7 mL Eppendorf tube with 0.2 mL F4 differentiation medium (one organoid per tube). 1,000 and 10,000 breast cancer cells were added to each organoid-containing tube and incubated for 24 h. For protocol two, individual organoids were transferred to an Ultra-low attachment 96-well round-bottom or flat-bottom plate (Corning Costar, NY) (one organoid per well). Excess medium was removed, and 1,000 stable GFP-expressing breast cancer cells were plated in each organoid-containing well (1,000 breast cancer cells/0.2 mL of F4 media per well). Plates were incubated at 37^o^C for 24 h with agitation. For both protocols, each organoid was subsequently washed in PBS and transferred to a clean well with 2 mL of F4 differentiation medium. Tumor-bearing organoids were maintained on an orbital shaker for up to 14 days at 37^o^C.

### Preparation of recombinant Lentivirus and infection of breast Cancer cells

The psPAX2 and pMD2G vectors and the pGIPZ lentiviral control vector expressing GFP were from Horizon Discovery (UK). HEK293 cells were transfected with 10 µg of pGIPZ lentiviral control vector, 10 µg of psPAX2, and 5 µg of pMD2G by the calcium phosphate method according to the instructions recommended by the manufacturer. Twelve hrs after transfection, the media were replaced with DMEM containing 5% FBS. The conditioned media were then collected twice at 1-day intervals and combined. After centrifugation and filtration with 0.22 μm filter, the supernatant was used to infect MCF-7, MDA-MB-231, HCC-1806, and SUM159PT breast cancer cells. The infected breast cancer cells were selected with 1 µg/mL puromycin in DMEM containing 10% FBS to obtain pools that stably expressed GFP. The pcPPT-mPGK-attR-sLPmCherry-WPRE vector was from Ximbio (London, England). The vector was used to make lentivirus for infection of MDA-MB-231 cells with stably expression of GFP. Double positive cells for both GFP and mCherry were selected through FACS and combined for experiments.

### Antibody

Primary antibodies used in this study were anti DCX (AB2253, Sigma, MO), EpCAM (MA5-12436, ThermoFisher Scientific, MA), GFP (2956, CST, MA), GFAP (3670, CST, MA), IBA1 (019-19741, Fujifilm, Japan), NG2 (AB5320, Sigma, MO), OLIG2 (AB9610, Sigma, MO), NEUN (MAB377, Sigma, MO), PCNA (SC9857, SCBT, CA), RFP (600-401-379, Rockland, PA), SOX2 (S9072, Sigma, MO), and TUJ1 (801,201, BioLegend, CA). Alexa fluorescence donkey anti-rabbit, Alexa fluorescence donkey anti-goat, fluorescein donkey anti-rabbit, fluorescein donkey anti-guinea pig, Alexa fluorescence donkey anti-mouse, fluorescein donkey anti-mouse (all from Jackson ImmunoRes, PA) were used as second antibodies.

### Histology, Immunofluorescence (IF), and TUNEL assay

Fixation of organoid-breast cancer cells was carried out for 16 h at 4 °C using 4% (w/v) freshly made, pre-chilled PBS-buffered paraformaldehyde (PFA). The organoids were embedded in paraffin, sectioned at 5 μm on a Leica microtome, essentially as we did before [81, 82]. Slides were stained with hematoxylin and eosin (H&E) for routine histological examination or left unstained for immunofluorescence (IF). H&E-stained sections were examined under a BX41 light microscope (Olympus America, Inc., Center Valley, PA), and images were captured with an Olympus digital camera (model DP70) using DP Controller software (Version 1.2.1.10 8). For IF, unstained tissues were first deparaffinized in 3 washes of xylene (3 min each) and then were rehydrated in graded ethanol solutions (100, 95, 70, 50, and 30%). After heat-activated antigen retrieval (Retriever 2000, PickCell Laboratories B.V., Amsterdam, Holland) according to the manufacturer’s specifications, sections were treated with Protein Block Serum Free (DAKO Corp, CA) at room temperature for 10 min. Slices were then incubated with the primary antibodies at 4 °C for 16 h in a humidified chamber, washed in PBS for 3 times (5 min each) and incubated with the 1:200 secondary antibodies for 1 h at room temperature. After incubation with secondary antibodies and washed in PBS for 3 times (5 min each), nuclei were stained with DAPI and mounted with Vectashield mounting medium (Vector Laboratories, CA). Digital photography was carried out as described previously [83].

Apoptotic cells were detected by TUNEL method according to the protocol provided by the manufacture within the In situ Cell Death Detection Kit-TMR Red (Roche, Germany).

### Statistical analysis

Statistical significance was evaluated by One-way ANOVA, Two-way ANOVA, and student’s t-test, with *p* < 0.05 as indicative of statistical significance using Graph Pad Prism (Version 7.0). The statistical differences between groups for limiting dilution of breast cancer cells in organoid were performed using ELDA as described previously [84]. The number of experiments used for quantification was indicated in the figure legends.

## Data Availability

The data and materials generated in this study are available upon request from the corresponding author.
